# Front Crawl Is More Efficient and Has Smaller Active Drag Than Backstroke Swimming: Kinematic and Kinetic Comparison Between the Two Techniques at the Same Swimming Speeds

**DOI:** 10.3389/fbioe.2020.570657

**Published:** 2020-09-24

**Authors:** Tomohiro Gonjo, Kenzo Narita, Carla McCabe, Ricardo J. Fernandes, João Paulo Vilas-Boas, Hideki Takagi, Ross Sanders

**Affiliations:** ^1^Department of Physical Performance, Norwegian School of Sport Sciences, Oslo, Norway; ^2^Institute for Sport, Physical Education and Health Sciences, The University of Edinburgh, Edinburgh, United Kingdom; ^3^Faculty of Health and Sport Sciences, University of Tsukuba, Tsukuba, Japan; ^4^Coaching of Sports and Budo, National Institute of Fitness and Sports in Kanoya, Kanoya, Japan; ^5^Faculty of Life and Health Sciences, Ulster University, Antrim, United Kingdom; ^6^Centre of Research, Education, Innovation and Intervention in Sport at Faculty of Sport, Porto Biomechanics Laboratory, University of Porto, Porto, Portugal; ^7^Faculty of Medicine and Health, The University of Sydney, Sydney, NSW, Australia

**Keywords:** aquatic locomotion, kinematics, kinetics, freestyle, back crawl

## Abstract

The purpose of this study was to investigate differences in Froude efficiency (η_*F*_) and active drag (*D*_*A*_) between front crawl and backstroke at the same speed. η_*F*_ was investigated by the three-dimensional (3D) motion analysis using 10 male swimmers. The swimmers performed 50 m swims at four swimming speeds in each technique, and their whole body motion during one upper-limb cycle was quantified by a 3D direct linear transformation algorithm with manually digitized video footage. Stroke length (*SL*), stroke frequency (*SF*), the index of coordination (*IdC*), η_*F*_, and the underwater body volume (*UWV*_*body*_) were obtained. *D*_*A*_ was assessed by the measuring residual thrust method (MRT method) using a different group of swimmers (six males) due to a sufficient experience and familiarization required for the method. A two-way repeated-measures ANOVA (trials and techniques as the factors) and a paired *t*-test were used for the outcomes from the 3D motion analysis and the MRT method, respectively. Swimmers had 8.3% longer *SL*, 5.4% lower *SF*, 14.3% smaller *IdC*, and 30.8% higher η_*F*_ in front crawl than backstroke in the 3D motion analysis (all *p* < 0.01), which suggest that front crawl is more efficient than backstroke. Backstroke had 25% larger *D*_*A*_ at 1.2 m⋅s^–1^ than front crawl (*p* < 0.01) in the MRT trial. A 4% difference in *UWV*_*body*_ (*p* < 0.001) between the two techniques in the 3D motion analysis also indirectly showed that the pressure drag and friction drag were probably larger in backstroke than in front crawl. In conclusion, front crawl is more efficient and has a smaller *D*_*A*_ than backstroke at the same swimming speed.

## Introduction

**FIGURE 1:**
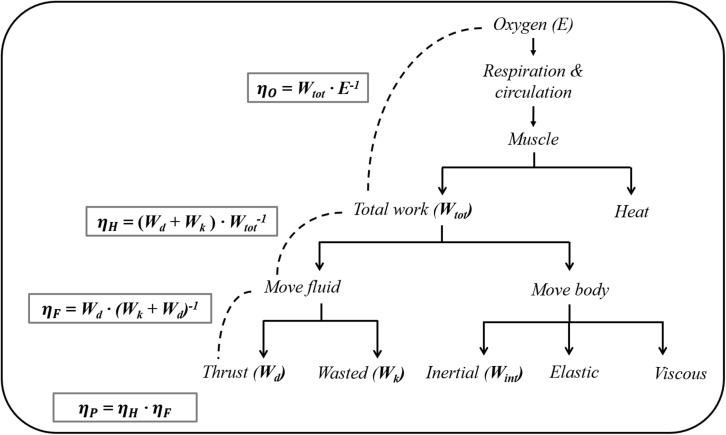
A diagram of the energy conversion and efficiency in swimming (adapted from Daniel, 1991). η_*O*_, overall efficiency; η_*P*,_ propelling efficiency; η_*H*_, hydraulic efficiency; η_*F*_, Froude efficiency.

Competitive swimming techniques are categorized into alternating (front crawl and backstroke) and simultaneous group (butterfly and breaststroke). Within the alternating techniques, swimmers usually achieve a faster swimming velocity (*v*) in front crawl than in backstroke despite their similarity such as six-beat kick during each upper limb cycle, probably due to the energy expenditure difference at a given *v* (energy cost; *C*). A lower *C* in front crawl than backstroke at 1.0, 1.2, 1.4, and 1.6 m⋅s^–1^ has been reported (Barbosa et al., 2006). However, this was based on different groups of swimmers and potentially affected by anthropometric and skill level differences. To overcome this limitation, *C* of the two techniques has been compared using the same swimmers, and 15% lower value in front crawl than in backstroke, despite the similar stroke frequency (*SF*) and stroke length (*SL*), has been reported (Gonjo et al., 2018). Mathematically, *C* is expressed as the equation below (Di Prampero et al., 1974; Zamparo et al., 2011).

(1) $$C=D_{A}\cdot {\left(\eta_{P}\cdot \eta_{O}\right)}^{-1}$$

where *D*_*A*_ is the hydrodynamic resistance the swimmer experiences when actively propelling in the water (active drag), η_*P*_ is the propulsive efficiency, and η_*O*_ is the gross efficiency, and this equation shows that an increase in η_*P*_ and/or a decrease in *D*_*A*_ contribute to low *C* (Zamparo et al., 2011). Therefore, the lower *C* in front crawl than backstroke suggests that the former technique has a higher η_*P*_ and/or a lower *D*_*A*_ than the latter at the same *v. η_*P*_* is the product of Hydraulic efficiency (η_*H*_) and Froude efficiency (η_*F*_) (Figure 1). η_*H*_ is affected by the internal power that is required to accelerate and decelerate the limbs relative to the center of mass (CM). The internal power is only 10–15% of the total mechanical power (Zamparo et al., 2005). Therefore, it is reasonable to assume that the primary factor determining η_*P*_ is η_*F*_ when conducting within-participant testing.

Since it is difficult to measure the total fluid and propulsive forces in swimming directly, methods of estimating η_*F*_ in both techniques are limited to mathematical models. The ratio of the mean *v* of CM (*v*_*CM*_) to the sum of the mean underwater three-dimensional (3D) speed of the left and right hands during the upper limb cycle has been suggested as an indicator of η_*F*_ (Figueiredo et al., 2013)—for the theoretical background of this approach, see Gonjo et al. (2018). This approach has been used for both front crawl and backstroke at the same *v*, and it has been suggested that the former technique is more efficient than the latter at 95% of the anaerobic threshold speed (Gonjo et al., 2018).

However, it is unclear if this is the case when swimming at a wide range of *v*. A negative within-participants correlation (*r* = −0.45, *p* = 0.01) between η_*F*_ and the index of coordination (the lag time between the propulsive motion of the left and right upper limbs as a percentage of the cycle time; *IdC*) in front crawl swimming has been reported (Figueiredo et al., 2013). This lag time varies from positive (left and right propulsive upper limb motions overlap each other) to negative (there is a gap between the propulsive motions) depending on *v* in front crawl (Chollet et al., 2000; Seifert et al., 2004), while it is negative regardless of *v* in backstroke (Chollet et al., 2008). Given the relationship between η_*F*_ and *IdC* and the difference in *IdC* between the techniques, the magnitude of η_*F*_ difference between the two techniques probably differs depending on *v*.

It should be noted that *IdC* is calculated differently depending on the swimming technique since the end of the propulsive motion is often defined as the hand exit from the water in front crawl (Chollet et al., 2000; Seifert et al., 2004), whereas in backstroke it is considered as the end of the second down-sweep motion (Chollet et al., 2008). In fact, when the end of backstroke propulsive motion was defined as the hand exit from the water (Schleihauf et al., 1988), an *IdC* value of 0.13% that is close to front crawl *IdC* has been observed (Lerda and Cardelli, 2003). Therefore, it is imperative to use the same motion phase definition in both front crawl and backstroke to assess the difference in *IdC* between the techniques at a wide range of *v* and its potential effect on η_*F*_.

Quantifying *D*_*A*_ in front crawl and backstroke is also very challenging due to the difficulty of measuring propulsive and resistive forces directly. It has been reported that the body frontal (cross-sectional) area perpendicular to the swimming direction is similar between front crawl and backstroke, and therefore the pressure drag (*D*_*p*_) of the two techniques is also similar assuming that the drag coefficient is 0.3 and constant (Gatta et al., 2015). However, it has been reported that it is more appropriate to use the wetted area as a reference area than the cross-sectional area for most of animal swimming forms except animals with a simple form and low Reynolds number (Alexander, 1990). The wetted area is difficult to assess directly; however, underwater body volume (*UWV*_*body*_) could be mathematically estimated from a 3D motion analysis (Yanai, 2001). Even though the surface area and volume are not the same concepts, these two variables should be strongly linked in a within-participant analysis. In other words, investigating *UWV*_*body*_ can be useful to indirectly investigate *D*_*A.*_

Besides the indirect approaches, there are three methods for assessing *D*_*A*_ that can be used for both front crawl and backstroke: the velocity perturbation and the assisted towing methods (Kolmogorov and Duplishcheva, 1992; Alcock and Mason, 2007) that only estimate *D*_*A*_ at the maximal effort of swimmers; and the measuring residual thrust (MRT) method (Narita et al., 2017) that can be used to quantify *D*_*A*_ in both front crawl and backstroke at controlled *v*. The MRT method is conducted in a flume with two wires attached to the swimmer’s body, which are connected to load cells at front and back of the flume, thereby fixing the swimmer at a certain location in the flume and measuring the force needed for the wires to fix the swimmer at the specific location (residual thrust). The swimmer is required to swim at nine different flow velocities without changing his/her motion, and *D*_*A*_ at the target velocity can be computed by establishing a regression curve plotting the residual thrust as a function of the flow velocity. The MRT method requires swimmers to have an adequate motor-skill to reproduce the same motion despite environmental (flow velocity) changes. Therefore, only swimmers who are familiar with a flume and the protocol can be tested. The accuracy of this method has not been established since obtaining the true active drag value during swimming is currently not possible due to a complex unsteady state of the water during swimming (Samson et al., 2017). However, the day-to-day variability of this method to assess *D*_*A*_ of the same swimmers was reported to be around 3.0–6.5% (Narita et al., 2017). This suggests that a difference in *D*_*A*_ between different techniques larger than approximately 6.5% can be considered as a meaningful result.

To summarize, it is currently unknown if *D*_*A*_ differs between front crawl and backstroke at the same speed despite the similar cross-sectional area of the body during the two techniques. There is evidence suggesting a higher η_*F*_ in front crawl than in backstroke at a low *v*, but it is unclear if the η_*F*_ differs across a wide range of *v* between the techniques. Therefore, the purpose of the present study was to investigate the differences in η_*F*_ and *D*_*A*_ using a 3D motion analysis and the MRT method. Based on the evidence provided in the extant literature, it was hypothesized that η_*F*_ would be higher in front crawl than in backstroke, and *D*_*A*_ would be similar between the two techniques.

## Materials and Methods

### 3D Motion Analysis

#### Participants

Participants for the 3D motion analysis were 10 male competitive swimmers (17.47 ± 1.00 years, 179.14 ± 5.43 cm, and 69.94 ± 6.54 kg), and their best records were 54.50 ± 1.23 and 60.56 ± 1.29 s in short course 100 m freestyle and backstroke, respectively. The participants regularly trained at least eight times per week, and the mean FINA point scoring for the best record in their specialized event was 600.20 ± 50.81 at the time of the data collection. Participants were informed about the procedures, benefits, and potential risks of the study (reviewed and approved by the ethics committee of the university based on the British Association of Sport and Exercise Sciences guidelines), and they (and a legal guardian for minors) provided written informed consent.

#### Testing Procedure

The testing session was conducted in a 25 m indoor pool and consisted of four 50 m trials for each technique with 83, 88, 93, and 100% of their backstroke maximum effort (83%BSv_max_, 88%BSv_max_, 93%BSv_max_, and 100%BSv_max_, respectively) for both techniques to compare outcome variables of front crawl and backstroke at the same *v*. The testing *v* was determined individually by a pilot study, and 83, 88, and 93% of the maximum *v* correspond to 400, 200, and 100 m *v* in front crawl according to a dataset provided in a previous study (Seifert et al., 2004). Throughout the testing, *v* was instructed by a visual light pacer (Pacer2, GBK-Electronics, Aveiro, Portugal) composed of a 25 m long cable with 26 LED lights for each meter from 0 to 25 m points. The pacer was located at the bottom of the pool for front crawl and attached to a stainless wire above the pool for backstroke.

The trials were recorded by six (four underwater and two above the water) digital video cameras (Sony, HDR-CX160E, Tokyo, Japan, with 50 fps sampling frequency, 1/120 s shutter speed, and 1,920 × 1,080/50 p movie resolution) that were synchronized using a LED system. The preparation for the participants and the testing lane calibration for the 3D motion analysis were conducted as previously described (Gonjo et al., 2018), and 3D coordinate data of 19 anatomical landmarks (the vertex of the head, the right and left of the: tip of the third distal phalanx of the finger, wrist axis, elbow axis, shoulder axis, hip axis, knee axis, ankle axis, fifth metatarsophalangeal joint, and the tip of the first phalanx) were obtained to calculate CM location of the body using manual digitizing with a sampling frequency of 25 Hz.

#### Data Processing and Analysis

Video files of each trial were trimmed in Ariel Performance Analysis System software (APAS: Ariel Dynamics, Inc., CA) so that one upper limb cycle (from a wrist entry to the subsequent entry of the same wrist) with five extra points before and after the cycle was included in the video files, which were extrapolated by reflection to an additional 20–30 points beyond the start and finish of the cycle. This strategy was to minimize errors associated with filtering and derivation of velocity data. However, it has been reported that 25 Hz digitizing with this strategy still causes a larger endpoint data distortion due to filtering compared with 50 Hz digitizing with 10 extra points (Sanders et al., 2015b). Therefore, the additional 20–30 points were individually adjusted for each swimmer and trial to minimize the distortion. The digitizing process was conducted with the APAS software, and a 4th order Butterworth filter with a 4 Hz cut-off frequency was applied for data smoothing.

Before calculating variables, the treated coordinate data were converted to 101 points representing percentiles of the stroke cycle time. CM location was determined by summing the moments of the segment CM about the X, Y, and Z right-hand reference axes (forward, upward, and lateral directions, respectively). Personalized body segment parameter data used for the CM calculation were obtained by the elliptical zone method (Jensen, 1978) with a digitizing method using a MATLAB program (Sanders et al., 2015a). *v*_*CM*_ was obtained by differentiating the X-displacement of CM over the whole stroke cycle by the time taken for the cycle. *SF* (cycles⋅min^–1^) was obtained as the inverse of the time that the swimmer took to complete one upper limb cycle, and *SL* (m⋅cycle^–1^) was obtained from the X-displacement of CM during the upper limb cycle (McCabe et al., 2011; McCabe and Sanders, 2012).

The wrist markers were assumed to represent the motion of the hands, and the mean 3D wrist speed during the underwater phase (*3Du*_*wrist*_: m⋅s^–1^) with the duration contains 101 samples was calculated by

$$3\,{\mathrm{Du}}_{\mathrm{wrist}}$$

(2) $$=\left(\sum_{k=1}^{100}\frac{\sqrt{{\left({\mathrm{dx}}_{k+1}-{\mathrm{dx}}_{k}\right)}^{2}+{\left({\mathrm{dy}}_{k+1}-{\mathrm{dy}}_{k}\right)}^{2}+{\left({\mathrm{dz}}_{k+1}-{\mathrm{dz}}_{k}\right)}^{2}}}{T_{\mathrm{interval}}}\right)\cdot 100^{-1}$$

Where *dx*, *dy*, and *dz* are X-, Y-, and Z-displacement of the wrist relative to CM, and *T*_*interval*_ is the time interval between each sample. In accordance with Figueiredo et al. (2011), η_*F*_ was then computed by

(3) $$\eta_{F}=v_{\mathrm{CM}}\cdot 3\,{\mathrm{Du}}_{\mathrm{wrist}}^{-1}$$

The mean volume of the body (*UWV*_*body*_) during one upper limb cycle was calculated by summing the volume of each segment in the water. The underwater segment volumes (*UWV*_*segment*_) of the head, upper-, and lower-limbs were calculated using the following equation, assuming that each segment was symmetrical around its long axis and has uniform density.

(4) $${\mathrm{UWV}}_{\mathrm{segment}}=V_{\mathrm{segment}}\cdot \left({\mathrm{UWL}}_{\mathrm{segment}}\cdot L_{\mathrm{segment}}^{-1}\right)$$

**FIGURE 2:**
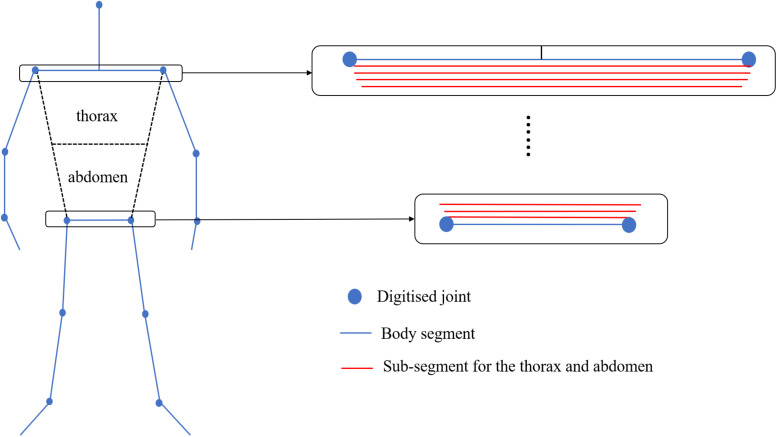
Sub-segments for the thorax and abdomen used to calculate the underwater volume of each part.

Where *V*_*segment*_ is the volume of the segment derived from the elliptical zone method, *UWL*_*segment*_ is the length of the segment under the water surface (Y-displacement = 0), and *L*_*segment*_ is the length of the segment. Since the thorax and abdomen are large segments where the rotation about the long axis cannot be ignored, a different approach was applied. For those segments, each segment was divided into 100 sub-segments (Figure 2), and the ratio of underwater volume to the total volume of thorax and abdomen was estimated by obtaining the sum of the underwater length of all sub-segments and calculating the ratio of it to the sum of the whole length of the sub-segments. The obtained ratio was then multiplied by the volume of thorax and abdomen acquired by the elliptical zone method to estimate the underwater volume of those segments.

Upper-limb bilateral coordination (the index of coordination: *IdC*) was also quantified as the lag time between left and right propulsive phases, which was from the beginning of the backward movement relative to the external reference frame until the wrist exit from the water (Figueiredo et al., 2013). Even though *IdC* has often been calculated differently between front crawl and backstroke (Chollet et al., 2000, 2008; Seifert et al., 2004), the same definition was applied to both techniques to compare *IdC* between the two swimming techniques with the same standard.

### MRT Method

#### Participants

The MRT method requires swimmers to perform in a flume and contains a complex testing protocol, as described in the section “Introduction.” In other words, sufficient experience in the testing environment and protocol is required for swimmers. Therefore, a different group of swimmers (who were experienced in both swimming in the flume and the protocol) from the 3D motion analysis were recruited for the MRT testing. The participants were six male national and international level competitive swimmers (21.50 ± 1.97 years, 175.83 ± 6.79 cm, and 69.17 ± 7.00 kg) whose 100 m long course best record for front crawl and backstroke were 52.95 ± 1.55 and 58.87 ± 3.33 s, respectively. The mean FINA point scoring of the participants was 760.82 ± 76.75, and they regularly completed at least nine training sessions per week at the time of the data collection. They had a minimum of 6 months of regular flume-swimming experience (including MRT testing protocol familiarization) and were specialized in either backstroke or the individual medley. The testing procedures and potential risks were explained to the participants, and each swimmer provided written informed consent.

#### Testing Procedure, Data Processing, and Data Analysis

After performing their individual warm-ups in an indoor pool, the testing was conducted in a flume with 5.5 m length, 2.0 m width, and 1.2 m depth (Igarashi Industrial Works Co. Ltd.) that the swimmers used in their regular training and testing. Two tri-axial load cells were positioned at the front and back of the flume, and swimmers were fixed at the center of the flume by two wires that were connected to the load cells, which measured the residual thrust produced by the swimmer toward the swimming direction. The target velocity was 1.2 m⋅s^–1^, which was the same for all participants to minimize any potential environmental differences among participants (e.g., the effects of the boundary layer between the flowing water and the wall/floor of the flume).

The MRT method is based on a least-squares quadratic curve fitting, meaning that more than three trials at different speeds should be conducted. Therefore, in addition to the target velocity trial, eight other trials (four with smaller and four with larger flow velocities than the target velocity) were assigned to swimmers to obtain an adequate curve fitting, i.e., the testing velocities were 1.00, 1.05, 1.10, 1.15, 1.20, 1.25, 1.30, 1.35, and 1.40 m⋅s^–1^. The target velocity was established by a pilot testing where swimmers could maintain their stroke kinematics at all nine flow velocity conditions (i.e., at velocities above 1.40 m⋅s^–1^, it was difficult for swimmers to maintain the same motion as the target velocity condition due to fatigue or the flow accelerating their upper limbs). *SF* of swimmers was controlled by a portable waterproof metronome (Tempo trainer Pro; FINIS, Inc., United States) during the nine trials to assist swimmers maintaining their stroke kinematics. To determine the guide *SF*, swimmers undertook one additional swimming trial with each swimming technique in the flume at the target velocity before the MRT trials. The *SF* during the pre-testing was obtained by video analysis and used as the guide *SF* at all nine trials.

The residual force swimmers produced (or experienced) was measured for 10 s at a sampling frequency of 50 Hz. Using the mean residual force at each flow velocity condition (1.00–1.40 m⋅s^–1^), *D*_*A*_ at 1.20 m⋅s^–1^ was estimated by obtaining the residual thrust at zero flow velocity using a least-square quadratic curve fitting. Since swimmers were supposed to maintain the same motion as they did at 1.20 m⋅s^–1^ in all nine trials, the estimated residual thrust at zero flow velocity was assumed to be equivalent to the mean propulsive and resistive forces at a free-swimming condition with the target velocity. More detail of the procedure is provided in the literature (Narita et al., 2017, 2018).

### Statistical Analysis

The normality of all datasets was checked and confirmed using the Shapiro-Wilk test. In the 3D motion analysis, a two-way repeated-measures ANOVA was used with the techniques and trials as two factors to assess the differences in *SF*, *SL*, η_*F*_, and *IdC* between the two techniques. Results corrected by the Greenhouse-Geisser procedure were used if the Mauchly’s sphericity assumption was violated (Field, 2007). When a significant interaction was observed in the two-way repeated-measures ANOVA test, simple main effect analysis was conducted using a paired *t*-test with the Bonferroni adjustment. In the MRT method, a paired *t*-test was used to compare *D*_*A*_ between front crawl and backstroke. Both analyses were conducted using IBM SPSS Statistics 24 (IBM Corporation, Somers, NY, United States), and statistical significance was set at *p* < 0.05.

## Results

In the 3D motion analysis, there were significant main effects of the techniques (*p* < 0.01) and trials (*p* < 0.05) in all variables (Table 1). *SF*, *SL*, η_*F*_, and *IdC* in front crawl were 3.5–7.7% lower, 5.9–11.9% longer, 28.6–33.7% larger, and 13.1–15.3% lower than in backstroke, respectively (Table 2), with no interaction between the techniques and trials. These results mean that swimmers achieved lower *SF*, longer *SL*, higher η_*F*_, and lower *IdC* in front crawl than in backstroke to achieve the same *v* regardless of its magnitude.

**TABLE 1:** *F*, *p*, and eta-squared (η^2^) values obtained from a two-way repeated-measures ANOVA for the kinematic variables.

	**Main effect**		**Interaction**
	**Techniques**	**Trials**	**Techniques × Trials**
*SF*	*F* = 14.69 (*p* < 0.01, η^2^ = 0.62)	*F* = 131.32 (*p* < 0.001, η^2^ = 0.94)	*F* = 0.66 (*p* = 0.59, η^2^ = 0.06)
*SL*	*F* = 16.40 (*p* < 0.01, η^2^ = 0.67)	*F* = 63.15 (*p* < 0.01, η^2^ = 0.86)	*F* = 1.25 (*p* = 0.31, η^2^ = 0.12)
*UWV*_*body*_	*F* = 110.67 (*p* < 0.001, η^2^ = 0.93)	*F* = 25.30 (*p* < 0.001, η^2^ = 0.74)	*F* = 3.40 (*p* < 0.05, η^2^ = 0.27)
η_*F*_	*F* = 125.45 (*p* < 0.001, η^2^ = 0.93)	*F* = 24.68 (*p* < 0.001, η^2^ = 0.73)	*F* = 1.68 (*p* = 0.20, η^2^ = 0.16)
*IdC*	*F* = 111.80 (*p* < 0.001, η^2^ = 0.93)	*F* = 19.21 (*p* < 0.001, η^2^ = 0.68)	*F* = 0.38 (*p* = 0.63, η^2^ = 0.04)

*SF, Stroke frequency; SL, Stroke length; UWV_*body*_, Underwater body volume; η_*F*_, Froude efficiency; IdC, The index of coordination.*

**TABLE 2:** Mean (Standard deviation) of the kinematic variables obtained by the three-dimensional motion analysis.

**Kinematic variables**		**83% BSv_max_**		**88% BSv_max_**		**93% BSv_max_**		**100% BSv_max_**	
Front crawl	*SF* (cycles⋅min^–1^)	29.38	(3.08)	31.34	(3.79)	34.64	(5.17)	42.67	(4.63)
	*SL* (m⋅cycle^–1^)	2.63	(0.20)	2.66	(0.22)	2.55	(0.25)	2.21	(0.16)
	*UWV*_*body*_ (liter)	60.54	(5.18)	60.15	(5.33)	60.08	(5.28)	59.35	(5.14)
	η_*F*_	0.47	(0.02)	0.46	(0.06)	0.44	(0.04)	0.41	(0.04)
	*IdC* (%)	–19.11	(1.76)	–16.68	(4.34)	–16.29	(3.13)	–12.72	(3.85)
Backstroke	*SF* (cycles⋅min^–1^)	30.44	(4.63)	33.24	(3.73)	37.53	(5.31)	44.81	(4.68)
	*SL* (m⋅cycle^–1^)	2.48	(0.22)	2.44	(0.20)	2.28	(0.22)	2.07	(0.17)
	*UWV*_*body*_ (liter)	62.67	(5.20)	62.69	(5.12)	62.38	(5.10)	62.08	(4.69)
	η_*F*_	0.35	(0.02)	0.35	(0.03)	0.34	(0.02)	0.32	(0.01)
	*IdC* (%)	–3.86	(4.09)	–2.43	(3.89)	–1.93	(4.31)	0.40	(5.31)

*BSv_*max*_, backstroke maximum velocity; SF, Stroke frequency; SL, Stroke length; UWV_*body*_, Underwater body volume; η_*F*_, Froude efficiency; IdC, The index of coordination.*

**FIGURE 3:**
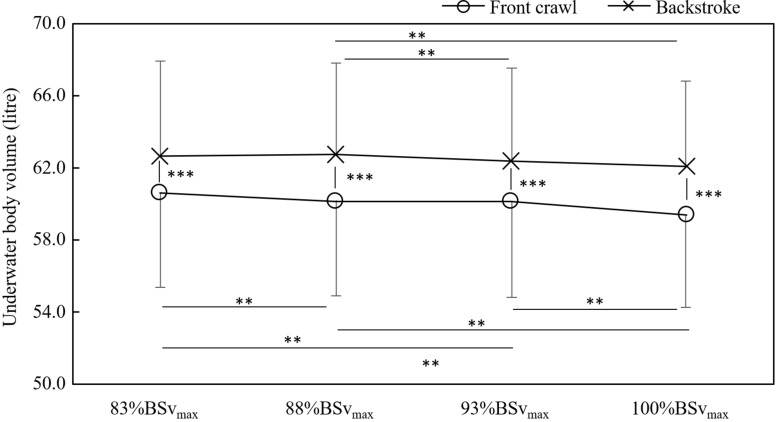
Differences in the underwater volume between each trial and technique (**p < 0.01 and ***p < 0.001).

On the other hand, there was an interaction between swimming techniques and trials in *UWV*_*body*_ with swimmers showing lower *UWV*_*body*_ in front crawl than in backstroke by 3.5–4.5% in all trials (all *p* < 0.001; Figure 3). In front crawl, *UWV*_*body*_ differed between each trial apart from 88%BSv_*max*_ vs. 93%BSv_*max*_. On the other hand, swimmers exhibited the differences only between 88%BSv_*max*_ vs. 93%BSv_*max*_ and between 88%BSv_*max*_ vs. 100%BSv_*max*_ in backstroke (Figure 3).

**FIGURE 4:**
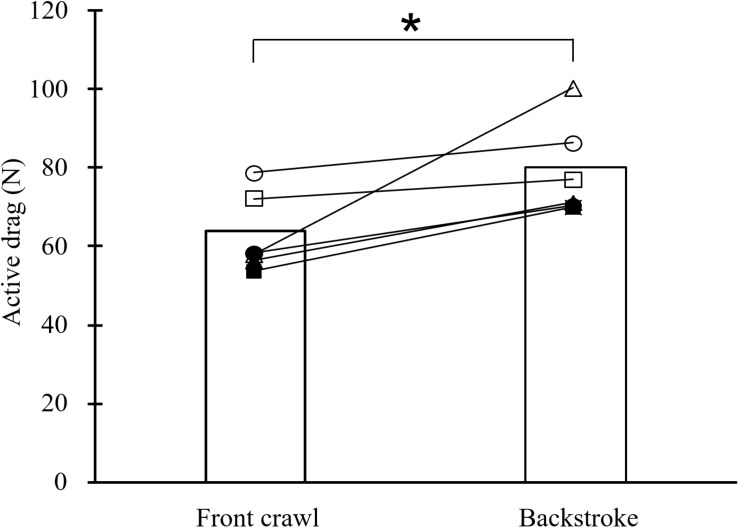
Mean and individual active drag in front crawl and backstroke obtained by the measuring residual thrust method (*p < 0.05).

In MRT testing, all swimmers showed higher *D*_*A*_ in backstroke than in front crawl with the average *D*_*A*_ among the swimmers being higher by 25% in backstroke (80.2 ± 12.1 vs. 64.1 ± 10.5 N; *p* < 0.05; Figure 4).

## Discussion

The purpose of this study was to assess the differences in η_*F*_ and *D*_*A*_ between front crawl and backstroke using a 3D motion analysis and the MRT method, testing two hypotheses; η_*F*_ would be higher in front crawl than backstroke; *D*_*A*_ would be similar between the two techniques. Counter to our second hypothesis, one of the main findings of the present study was higher *D*_*A*_ in backstroke than in front crawl, which was indirectly and directly supported by both 3D motion analysis and the MRT method, respectively. In swimming, *D*_*A*_ can be explained by *D*_*p*_, the wave drag (*D*_*w*_), and the friction drag (*D*_*f*_), and the primary source of *D*_*A*_ is *D*_*p*_ (Pendergast et al., 2006). The magnitude of the total drag is determined by the drag coefficient, water density, reference area and *v*, and the reference area particularly affects *D*_*p*_ as this drag component largely depends on the shape and size of the body in the water (Alexander, 1990).

In swimming research, the cross-sectional area has often been used as the reference area, and it has been suggested that *D*_*A*_ is similar between front crawl and backstroke if the drag coefficient and *v* are identical because the cross-sectional area is similar between the two techniques (Gatta et al., 2015). However, it has been reported that use of the cross-sectional area in most of animal swimming forms is inappropriate because the shape of many swimming animals is too complex to assume the cross-sectional area as the reference area (Alexander, 1990). In the 3D motion analysis, *UWV*_*body*_ in backstroke was larger than that in front crawl. Given the impact of the definition of the surface area on *D*_*p*_ and that the wetted area is more suitable as the reference area than the cross-sectional area in animal swimming (Alexander, 1990), the difference in *UWV*_*body*_ indirectly suggests the possibility of distinct *D*_*p*_ between front crawl and backstroke.

The difference in *UWV*_*body*_ also suggests a possibility of distinct *D*_*f*_ between front crawl and backstroke. *D*_*f*_ is determined by the roughness of the body surface that is exposed to the water (Marinho et al., 2009). The larger *UWV*_*body*_ in backstroke than in front crawl implies that a larger area of the body was in the water in backstroke than in front crawl. Therefore, *D*_*f*_ in backstroke might have also been greater compared with front crawl. During swimming on the water surface, *D*_*A*_ is also affected by *D*_*w*_, which is increased with almost the cube of *v* (Vennell et al., 2006), and it has been reported that *D*_*w*_ is critical over 1.7 m⋅s^–1^ (Toussaint, 2002). However, *D*_*A*_ was assessed at a much slower speed than 1.7 m⋅s^–1^ in the present study; consequently, it is reasonable to conclude that the other drag components (*D*_*p*_ and *D*_*f*_) were the primary determinants of *D*_*A*_ in the current study.

Adding to the indirect evidence from the 3D motion analysis suggesting higher *D*_*A*_ in backstroke than in front crawl, the result from the MRT analysis clearly shows that front crawl has less *D*_*A*_ than backstroke. In the MRT method, the result only shows the total drag, and the drag components cannot be obtained. However, given that the tested speed is low (1.2 m/s) where the wave drag effect on the total drag is small (Vennell et al., 2006), it is likely that the difference was either/both due to distinct *D*_*p*_ or/and *D*_*f*_ between the techniques.

Since the 3D motion analysis and the MRT analysis were conducted using a different group of swimmers, it is difficult to link the information obtained from the two analyses. However, the present study was focusing on differences in a within-participants factor (techniques) rather than a between-participants factor (swimmers), and both groups directly or indirectly showed higher *D*_*A*_ in backstroke than in front crawl. This is an important fact that different testing settings with different groups of swimmers both suggested the same conclusion, which strengthened the probability of the difference in *D*_*A*_ between the techniques.

**FIGURE 5:**
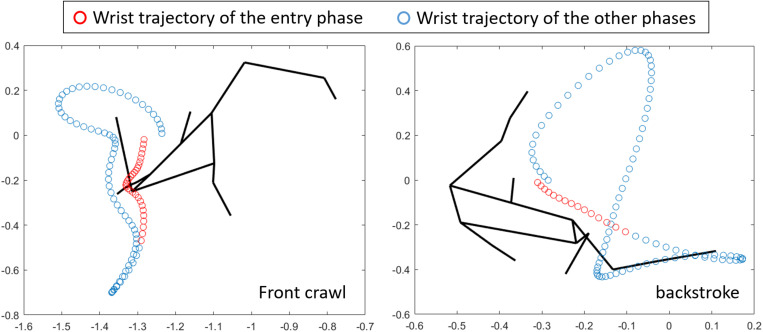
Frontal view of a whole-body stick figure and the wrist trajectory in front crawl and backstroke.

Given that the cross-sectional area is not different between front crawl and backstroke (Gatta et al., 2015), the difference in *UWV*_*body*_ was probably due to the body alignment rather than the position of the entire body relative to the water surface. One potential explanation is that the position of the head and shoulder might be higher in front crawl than backstroke due to the hydrodynamic force produced by the downward motion of the hand at the beginning of the stroke. Figure 5 shows the wrist trajectory of a participant in front crawl and backstroke from the frontal view as an example. During the period between hand entry and the beginning of the backward movement of the hand relative to the external reference frame (entry phase), the primary hand motion in front crawl is downward, whereas sideways motion is dominant in backstroke. Because of this difference, it is likely that the upward component of the hydrodynamic force was greater in front crawl than in backstroke and resulted in the difference in *UWV*_*body*_ between the techniques. However, this hypothesis needs to be further investigated to establish the relationships between the hand trajectory, hydrodynamic forces, body alignment, and *UWV*_*body*_.

It has been reported that front crawl and backstroke have similar *SF* and *SL* at a low swimming velocity (Gonjo et al., 2018). The similar *SF* and *SL* reported in the previous study are in conflict with the present study (higher *SF* and shorter *SL* in backstroke than in front crawl), which can be explained by the potential difference in *D_*A*_.* In the present study, the testing speed was about 20–45% higher than Gonjo et al. (2018), who tested swimmers below the anaerobic threshold. Since *D*_*A*_ increases with the square or cube of the swimming velocity (Barbosa et al., 2010; Narita et al., 2017), the difference in *D*_*A*_ between the techniques should also become large in a high swimming velocity condition. Consequently, *D*_*A*_ in Gonjo et al. (2018) might not have been so critical that did not produce differences in *SF* and *SL*, whereas the effect was likely much larger in the present study compared with the previous study.

In the 3D motion analysis, η_*F*_ exhibited significant main effects of the technique and trial without a significant interaction, which suggested that front crawl is more efficient than backstroke regardless of the magnitude of *v*, and therefore the first hypothesis was supported. Considering the probable difference in *D*_*A*_ between the techniques and the result of η_*F*_, it is likely that swimmers have a higher energy cost in backstroke than in front crawl since the energy cost is positively and negatively related to the work required to overcome the drag and η_*F*_, respectively (Di Prampero et al., 1974; Zamparo et al., 2011). In other words, the energy cost in backstroke is probably higher than in front crawl due to a dual effect of larger *D*_*A*_ and lower η_*F*_. The possibility of higher energy cost in backstroke than in front crawl is also supported by Gonjo et al. (2018) who reported a distinct energy cost between the techniques at *v* below the anaerobic threshold.

In the present study, swimmers had higher *IdC* in backstroke than in front crawl. From the slowest to fastest trials, swimmers increased *IdC* by 6.4 and 4.2% in front crawl and backstroke, respectively, without an interaction effect (techniques × trials). These results suggested that swimmers increase their *IdC* when incrementing their *v* in both techniques, with backstroke always exhibiting higher *IdC* at the same *v*, meaning that backstroke had a shorter gap in time between the left and right propulsive motion. This was counter to our expectation since the extant literature reported higher *IdC* in front crawl than in backstroke (Seifert et al., 2004; Chollet et al., 2008). The *IdC* calculated in the present study was generally lower in front crawl, and higher in backstroke, than *IdC* presented in the literature.

The difference in *IdC* between the present study and the literature in front crawl was probably due to the difference in the method of quantifying the coordination. In the current study, *IdC* was obtained using the upper limb kinematics based on the external reference frame, whereas many studies using *IdC* to assess inter-limb coordination use the video observation, sometimes using panning video footage, without obtaining the global coordinates. Swimmers start moving their hand backwards relative to their body before the hand starts traveling backwards relative to the water due to the forward body motion. Therefore, the propulsive phase duration might be shorter (due to the distinct point of the beginning of the phase) in the definition using the external reference frame than that using the video observation, thereby affecting the underestimation of *IdC*.

The opposite tendency in the difference between the present study and the literature in backstroke was probably due to the distinct definition of the end of the propulsive motion. In the extant literature, the propulsive motion in backstroke has been considered to finish at the end of the second down-sweep motion (Chollet et al., 2008). On the other hand, the present study defined the end of the propulsive motion as the wrist exit. Therefore, the propulsive phase in the present study is likely to be longer than in the other studies, and consequently, the time gap between the left and right propulsive motion is shorter than the previous studies. In fact, a previous study (Lerda and Cardelli, 2003) used a similar definition as the current study and reported *IdC* value of 0.13% at *v* corresponding to 50 m race, which is comparable to the *IdC* in the present study.

Since the definition of *IdC* in this study differs from that in many other studies, it is not appropriate to compare the absolute *IdC* value obtained in the current study with that in the literature. However, the present study used the same definition as Figueiredo et al. (2013), who reported that *IdC* is inversely correlated with η_*F*_. This evidence supports the possibility that the decrease in η_*F*_ was partly due to the increase in *IdC* in both swimming techniques, and the higher η_*F*_ in backstroke than in front crawl could also be explained by the difference in *IdC*. More specifically, the larger *IdC* contributed to the higher *SF* in backstroke than in front crawl as those variables are positively associated (Chollet et al., 2000, 2008), which resulted in the lower η_*F*_ in backstroke since *SF* and η_*F*_ have an inverse relationship when the upper-limb motion is described as a simplified model (Zamparo et al., 2005).

The present study has three limitations. The first limitation was the lack of the link between the lower limb kinematics and η_*F*_. Even though swimmers perform similar lower limb motion (six flutter kicks) in both front crawl and backstroke, the mechanism of the kicking might differ between the techniques due to the distinct ventral and dorsal posture. Nevertheless, the effect of lower limbs on the η_*F*_ results in the current study should be minor since the net contribution of the kicking to propulsion is small (about 15%) and similar between the techniques (Bartolomeu et al., 2018).

The second limitation is the assumption that swimmers can maintain their motion when controlling *SF* in the MRT method. The MRT method is based on several trials with different flow velocity in a flume, and yet swimmers should maintain a given motion and *SF* to calculate *D*_*A*_. It is possible that swimmers slightly change their relative duration of the underwater and recovery phases even if they maintain a required *SF* because of the changes in the flow velocity. However, this study used the same flow velocity conditions in both front crawl and backstroke, and the error due to the task (maintaining the motion with different flow velocity condition) should be systematic and of similar magnitude in front crawl and backstroke. This means that even if the absolute *D*_*A*_ values in the present study contain systematic errors, the effect of the error on the magnitude of the difference in *D*_*A*_ between the two techniques should be small. In fact, the difference in *D*_*A*_ between the two techniques in the present study (25%) was much larger than the test-retest error (3.0–6.5%) reported in the literature (Narita et al., 2017).

The third potential limitation is the sample size (ten and six swimmers in the motion analysis and the MRT method, respectively). Small sample size does not affect the type I error possibility but increase the risk of a type II error (Harmon and Losos, 2005), which is the probability of incorrectly accepting the null hypothesis. Thus, any results that do not show statistical difference or effect should be treated carefully when testing with low sample size. However, in the current study, all non-significant results showed *p*-value far from the alpha level (*p* ≥ 0.20), and it is unlikely that some results were incorrectly interpreted as non-significant.

## Conclusion

In conclusion, swimmers can swim more efficiently with smaller *D*_*A*_ in front crawl than in backstroke at the same *v*. Front crawl also has longer *SL*, lower *SF*, and smaller *IdC* than backstroke at the same *v* up to backstroke maximum speed. Detailed causes of the difference in *D*_*A*_ between the two techniques and potential differences in the lower limb kinematics and its effect on the performance should be further investigated. The findings of the current study imply that backstroke is more physically demanding than front crawl swimming. Coaches should consider this difference between the two techniques when prescribing training to front crawl and backstroke swimmers (e.g., prescribing lower intensity or volume for backstroke swimmers) to avoid overtraining.

## Data Availability Statement

The raw data supporting the conclusions of this article will be made available by the authors, without undue reservation, to any qualified researcher.

## Ethics Statement

The studies involving human participants were reviewed and approved by the ethics committee of the University of Edinburgh, the ethics committee of Porto University, and the ethics committee of the University of Tsukuba. Written informed consent to participate in this study was provided by the participants and their legal guardian/next of kin for minors.

## Author Contributions

TG and RS developed the concept of the research. TG, CM, and RS designed the initial experimental setting for the 3D motion analysis that RF and JV-B further extended. TG, RF, and JV-B recruited participants and conducted data collection for the 3D motion analysis. TG, CM, and RS performed data treatment and analysis for the 3D motion analysis. KN and HT developed the theory and computation for the MRT method, recruited participants, and conducted data collection and analysis for the MRT analysis. TG wrote the first draft of the manuscript with support from KN. All authors contributed to the article and approved the submitted version.

## Conflict of Interest

The authors declare that the research was conducted in the absence of any commercial or financial relationships that could be construed as a potential conflict of interest.
